# Partnership and fatherhood outcomes in young men diagnosed with cancer: a nationwide register-based study

**DOI:** 10.1007/s11764-025-01849-7

**Published:** 2025-06-10

**Authors:** Line Bentsen, Helle Pappot, Kirsten Tryde Macklon, Lone Schmidt, Ditte Vassard

**Affiliations:** 1 Department of Oncology, Copenhagen University Hospital - Rigshospitalet, Copenhagen, Denmark; 2 Institute of Clinical Medicine, University of Copenhagen, Copenhagen, Denmark; 3 Fertility Clinic, Department of Gynaecology, Fertility and Childbirth, Copenhagen University Hospital - Rigshospitalet, Copenhagen, Denmark; 4 Section of Social Medicine, Department of Public Health, University of Copenhagen, Copenhagen, Denmark

**Keywords:** Cancer, Young adults, Infertility, Fatherhood, Epidemiology

## Abstract

**Purpose:**

A cancer diagnosis during young adulthood can impact identity, relationships, and family building. While young men with cancer face fertility risks, data on fatherhood and partnership outcomes remain limited. This study aimed to examine the hazard ratios (HRs) for fatherhood and partnership formation in young men diagnosed with cancer compared to age-matched comparisons.

**Methods:**

This nationwide register-based study, based on the DANAC II cohort, included men diagnosed with cancer at 18–39 years (1978–2016) and age-matched male comparisons. Cox regression models estimated HRs of fatherhood and partnership formation, adjusting for time of diagnosis or study entry, immigration status, and pre-diagnosis children.

**Results:**

Among 16,913 men with cancer and 1,353,040 comparisons, those with cancer had a lower HR of fatherhood (0.88 [95% confidence interval (CI) 0.86–0.91]), the lowest among men with lymphoma, central-nerve system (CNS), and gastrointestinal cancers. Fatherhood probabilities improved over time, with older men showing the greatest gains. Among men who were single at diagnosis or study entry, the HR for partnership formation was similar to the comparison group (1.03 [95% CI 0.99–1.07]), except for those with gastrointestinal or CNS cancers.

**Conclusions:**

Fatherhood HRs were generally lower than in comparisons, varying by cancer group, age, and diagnosis period. However, single men diagnosed with cancer typically formed partnerships similar to the comparisons.

**Implications for Cancer Survivors:**

The lower probability of fatherhood highlights the need for and importance of oncofertility counseling, timely fertility preservation referrals, and follow-up care tailored to cancer groups and age at diagnosis.

**Supplementary Information:**

The online version contains supplementary material available at https://doi.org/10.1007/s11764-025-01849-7.

## Introduction

A cancer diagnosis during young adulthood can significantly impact various aspects of life, including identity development, education, sexual health, and family building [1–3]. For young adult men, cancer and its treatments, such as chemotherapy, radiation, and surgery, can pose risks to sexual and reproductive health. These risks include reduced sperm function and count, as well as erectile dysfunction and ejaculation deficits [4–8]. Beyond physiological effects, the psychological burden of a cancer diagnosis can complicate romantic relationships due to the associated stress, anxiety, and potential stigma, often impacting self-esteem and social interactions [9–11]. Recent psychosocial-oncology research indicates that treatment-related physiological sequelae (e.g., gonadotoxicity) and cancer-related psychological distress act synergistically, jointly shaping young male survivors’ reproductive decision-making and their ability to initiate and sustain intimate relationships.

The risk of reduced ability to have children and entering a romantic relationship are critical concerns for young adult cancer patients, significantly influencing their health-related quality of life [12–18]. As cancer treatments can lead to temporary or permanent infertility, advancements in semen cryopreservation have expanded options for future family building [19, 20]. Despite internationally recognized cancer-related fertility preservation guidelines [20–22], previous studies suggest that male cancer survivors are less likely to become fathers compared to the general population. To date, only a few studies have examined fatherhood among male survivors, of which most include childhood and adolescent cancer survivors. Syse et al. (2007), who included males diagnosed between ages 17 and 44, showed an odds ratio (OR) of 0.76 (95% CI 0.72–0.79) of fathering a first child after cancer [23]. Tang et al. (2016) included men diagnosed before age 70 and reported a standardised birth rate (SBR) of 0.80 (95% CI 0.78–0.82) [24]. Murphy et al. (2025) found that among the 42,896 male AYAs aged 15–39 years when diagnosed with cancer, the 10-year cumulative incidence of live birth after diagnosis ranged from 10% for male AYAs with gastrointestinal cancers to 28% for male AYAs with thyroid cancer [25]. Also, Patel et al. (2022), who explored a first live birth for female partners of males (≥ 18 years) after a cancer diagnosis, found a 31% lower fertility rate compared to men without cancer (hazard ratio (HR), 0.69; 95% CI, 0.65–0.72) [26]. Gunnes et al. (2016), who included males with cancer and comparisons before the age of 25, also reported a lower HR (0.72 [95% CI 0.68–0.76]) of fatherhood after diagnosis among male cancer survivors compared to men without cancer. Furthermore, they found lower marriage HRs among cancer survivors (0.93 [95% CI 0.86–1.00]), especially among CNS cancer survivors [27]. Similarly, Armuand et al. (2017) included childhood or adolescent cancer survivors diagnosed between ages 0 and 21 and found a lower adjusted HR (0.68 [95% CI 0.59–0.79]) of fathering a first child compared to comparisons [28].

The incidence of cancer types in young adults differs from those in childhood. While childhood cancers predominantly involve hematopoietic and lymphoid malignancies, CNS tumors, embryonal tumors, and sarcomas, young adults are diagnosed with a broader range of cancers including testicular cancer, malignant melanoma, gastrointestinal cancers, leukemia, and lymphoma [29].

While substantial research highlights lower parenthood rates, especially among childhood and adolescent cancer survivors [27, 28, 30, 31], data on fatherhood and partnership formation among young adult men with cancer across age and cancer groups remains somewhat dated and limited. Thus, this study aims to take a life-course perspective on fatherhood and partnership after cancer. We compare young men diagnosed with cancer between ages 18 to 39 to age-matched undiagnosed men in the background population to (1) estimate the HR for becoming a father after diagnosis and (2) estimate the HR of forming a partnership after diagnosis among men who were single at the time of diagnosis.

## Materials and methods

### Study design and setting

This study employed a matched cohort design utilizing nationwide, register-based data from the Danish National Assisted Reproductive Technology-Couple (DANAC II) Cohort.

The DANAC II cohort encompasses the Danish population, comprising individuals born between 1948 and 2001, totaling approximately 3.1 million people. For this study, the population of interest included men diagnosed with cancer and an age-matched male sample of the general population. The date of cancer diagnosis or a comparable age-matched inclusion date for the comparison group served as the reference point. This was used to assess (1) the time until fathering a first child after cancer diagnosis or study entry and (2) the time until entering a marital or cohabiting partnership among men who were single at diagnosis or study entry. Follow-up began at the date of diagnosis or matching (for comparisons) and ended with the occurrence of the outcome, at death, emigration, or the end of follow-up (December 31, 2017), whichever occurred first.

### Study Participants

The study population included men diagnosed with cancer between the ages of 18 and 39 during the period 1978 to 2016. The age range is adopted by the US National Cancer Institute for adolescents and young adults (AYA)[32]. We matched each cancer-diagnosed individual with 80 men from the general population who were alive, of the same birth year, and cancer-free at the time of matching. Using the nationwide registers, we were able to select this high level of age-matched controls per case, maximizing the size and representativeness of the comparison group without increasing the analytical effort relative to smaller ratios. Age was chosen as the sole matching variable because it is the dominant determinant of male fertility and family-formation timing, whereas matching on additional characteristics could cause over-matching and would unnecessarily restrict the control pool [33]. We included only the first recorded cancer diagnosis. Comparisons were censored if they were later diagnosed with cancer and were then age-matched with comparisons without cancer.

### Variables

The primary exposure variable was a cancer diagnosis, defined as a binary variable (yes/no). We further grouped the cancer diagnoses coded by the International Classification of Diseases (ICD-10) [34] into the following categories: leukemia, lymphoma, CNS cancers, gastrointestinal cancers, malignant melanoma, testicular cancer, and other solid tumors (including rarer sites such as thyroid, sarcoma, lung, urothelial, and the < 3 male breast-cancer cases [C50]). We based the diagnostic groups on similarities in disease characteristics and treatments, with smaller groups combined to ensure sufficient statistical power (see Supplementary 1).

For baseline characterization, the study population was stratified into age groups (18–25, 26–32, and 33–39 years at diagnosis or study entry), reflecting typical family formation ages in Denmark [35]. Additionally, periods of diagnosis or study entry were categorized as follows: 1978–1989, 1990–1999, 2000–2009, and 2010–2016. Immigration status was classified as Danish origin, immigrant, or second-generation immigrant. Partnership status was categorized as single, married, or cohabiting. Educational attainment was categorized according to the Danish education system: primary school, high school, vocational training, and short-, medium-, or long-cycle higher education.

### Data sources

We obtained data from Danish national registries, with the cancer diagnoses sourced from the Danish Cancer Register [36], live birth records from the Danish Medical Birth Register [37], and mortality data from the Register of Causes of Death [38]. Additionally, information on education, migrations, and partnership status was collected from Statistics Denmark, available from 1985 onward [39].

### Statistical methods

Confounders and mediators were determined using directed acyclic graphs (DAGs) and included variables assessed at diagnosis or matching [40]. For analyses of **fatherhood**, potential confounders included age, year of study entry, immigration status, and existing, registered children prior to study entry (see Supplementary 2). Calendar year of index date, immigration status, and pre-diagnosis parity (none vs ≥ one child) were measured at study entry and modeled as fixed covariates, while age served as the underlying timescale so that age varied implicitly throughout follow-up. Partnership formation was considered an effect modifier in analyses exploring fatherhood outcomes. For analyses of **partnership formation**, potential confounders included age at study entry, year of study entry, and immigration status (see Supplementary 3).

Educational attainment and partnership status were considered mediators rather than confounders, given the relatively late age at which education is typically completed and stable partnerships are formed in Denmark. Therefore, these variables were excluded from the primary confounder adjustments.

Baseline sociodemographic characteristics were summarized descriptively. Differences in follow-up time, mainly influenced by higher mortality rates among cancer patients, were illustrated using cumulative incidence curves, illustrating death as a competing event.

Cox proportional hazards regression models were used to estimate HRs and 95% CIs, comparing the probability of fathering a live-born child post-diagnosis between young men diagnosed with cancer and age-matched comparisons, and the probability of entering a marital or cohabiting relationship among men who were single at diagnosis and age-matched comparisons.

Age served as the underlying timescale in the models, inherently adjusting for its effect. In analyses including age and cancer groups, men with cancer were consistently compared to age-matched comparisons within the same strata. Follow-up time was summarized using median values and interquartile ranges (IQR). We assessed temporal trends in fatherhood post-diagnosis or study entry through separate analyses for each decade.

Sensitivity analyses excluded men whose partners were presumed pregnant at the time of diagnosis or matching, defined as live births occurring within 259 days of study entry. Missing data on educational attainment and partnership status were concentrated in the early study period (1978–1985) and were assessed as missing at random based on sensitivity analyses. We excluded missing values from analyses involving these variables. However, because these variables were not included in the primary models, their overall impact was minimal (e.g., only 5% missing for immigration status). We conducted sensitivity analyses to check for robustness for changes in periods (5-year intervals).

Assumptions of proportional hazards were validated using Kaplan–Meier plots.

All statistical analyses were performed using SAS version 9.4 (SAS Institute, Cary, NC, USA).

## Results

Table 1 presents the baseline sociodemographic characteristics of the study population consisting of 16,913 men diagnosed with cancer and 1,353,040 age-matched male comparisons from the background population. The median age at cancer diagnosis or study entry was 31.7 years (IQR 26.6–36.0). Distribution in age intervals showed an increased frequency of men diagnosed with cancer with increased age. Among the cancer groups, men with testicular cancer had the highest frequency, accounting for 6438 cases (38% of the total cancer cohort), while men with lymphoma had the lowest frequency, with 741 cases (4%).

**Table 1:** Baseline sociodemographic characteristics for the study population including men diagnosed with cancer when aged 18–39 (*n* = 16,913) and the age-matched, undiagnosed comparisons from the background population (*n* = 1,353,040) from 1978 to 2016. The men with cancer are stratified into cancer groups.* CNS *central-nerve system, *IQR* interquartile range

	All	Comparisons	Leukemia	Lymphoma	CNS	Gastro-intestinal	Testicular	Melanoma	Other
Cases of men with cancer or undiagnosed comparisons: n (%)	16,913 (100)	1,353,040 (100)	862 (5)	741 (4)	1080 (6)	1278 (8)	6438 (38)	2257 (14)	4257 (25)
Age at cancer diagnosis or study entry: median years (IQR)	31.7 (26.6–36.0)	31.7 (26.6–36.0)	31.5 (24.6–35.8)	31.0 (24.9–35.9)	31.9 (26.5–36.4)	34.8 (30.4–37.8)	30.3 (26.0–34.6)	33.0 (28.1–36.7)	32.3 (26.5–36.6)
Age intervals: n (%) • 18–25 years • 26–32 years • 33–39 years	3822 (23) 5854 (35) 7237 (42)	306,414 (23) 467,381 (35) 579,245 (42)	249 (29) 261 (30) 352 (41)	224 (30) 210 (28) 307 (42)	243 (23) 369 (34) 468 (43)	118 (9) 368 (29) 792 (62)	1614 (25) 2601 (40) 2223 (35)	381 (17) 750 (33) 1126 (50)	993 (23) 1295 (30) 1969 (46)
Immigration status at diagnosis or study entry: n (%)									
Danish origin Immigrant Second generation immigrant Missing	15,178 (93) 131 (1) 1046 (6) 558	1,000,922 (83) 10,689 (1) 191,592 (16) 149,837	695 (89) 8 (1) 78 (10) 81	629 (85) 16 (2) 92 (13) 4	930 (92) 9 (1) 70 (7) 71	1047 (88) 21 (2) 125 (10) 85	6089 (96) 36 (1) 239 (3) 74	2140 (96) 11 (1) 82 (4) 24 1	3648 (90) 30 (1) 360 (9) 219
Educational level at diagnosis or study entry: n (%)									
Primary school High school Vocational education Short-cycle higher education Medium-cycle higher education Long-cycle higher education Missing	3898 (28) 1618 (12) 5291 (38) 609 (4) 1481 (11) 1066 (8) 2947	255,302 (28) 98,820 (11) 346,701 (39) 39,088 (4) 91,683 (10) 64,611 (7) 456,835	241 (36) 66 (10) 214 (32) 31 (5) 63 (9) 53 (8) 194	200 (30) 97 (14) 198 (29) 30 (4) 82 (12) 67 (10) 67	267 (30) 96 (11) 330 (37) 26 (3) 99 (11) 67 (8) 195	323 (32) 87 (9) 410 (40) 41 (4) 92 (9) 67 (7) 258	1428 (27) 636 (12) 2161 (40) 235 (4) 538 (10) 375 (7) 1065	386 (19) 271 (13) 733 (36) 112 (6) 303 (15) 232 (11) 220	1053 (32) 365 (11) 1245 (38) 134 (4) 307 (9) 205 (6) 948
Partnership status at diagnosis or study entry: n (%)									
Married Cohabiting Single Missing	5444 (37) 3557 (24) 5725 (39) 2187	372,104 (38) 235,065 (24) 365,351 (38) 380,520	307 (42) 152 (21) 265 (37) 138	238 (33) 168 (23) 325 (44) 10	393 (42) 211 (22) 334 (36) 142	451 (40) 234 (21) 434 (39) 159	1867 (34) 1505 (27) 2191 (39) 875	866 (41) 547 (26) 684 (33) 160	1322 (37) 740 (21) 1492 (42) 703
Men with one or more children at diagnosis or study entry: n (%)	7239 (43)	538,486 (40)	357 (41)	263 (35)	496 (46)	674 (53)	2464 (38)	1150 (51)	1835 (43)
Men without children at diagnosis or study entry: n (%)	9674 (57)	814,554 (60)	505 (59)	478 (65)	584 (54)	604 (47)	3974 (62)	1107 (49)	2422 (57)

In total, 93% of men with cancer were of Danish origin, while immigrants and second-generation immigrants constituted 1% and 6% of the men diagnosed with cancer, respectively. The control group had a slightly higher proportion of second-generation immigrants (16%). No differences in educational level or partnership status at diagnosis or study entry were found between men with cancer and comparisons. Approximately 37% were married, 24% were cohabiting, and 39% were single at the time of diagnosis or study entry.

In total, 43% of men with cancer and 40% of comparisons had one or more children before diagnosis or study entry. Those with gastrointestinal cancers (53%) and malignant melanoma (51%) were more likely to have one or more children at diagnosis than those with leukemia (41%), testicular cancer (38%), or lymphoma (35%).

### The cumulative incidence of fatherhood and death after diagnosis

**Fig. 1:**
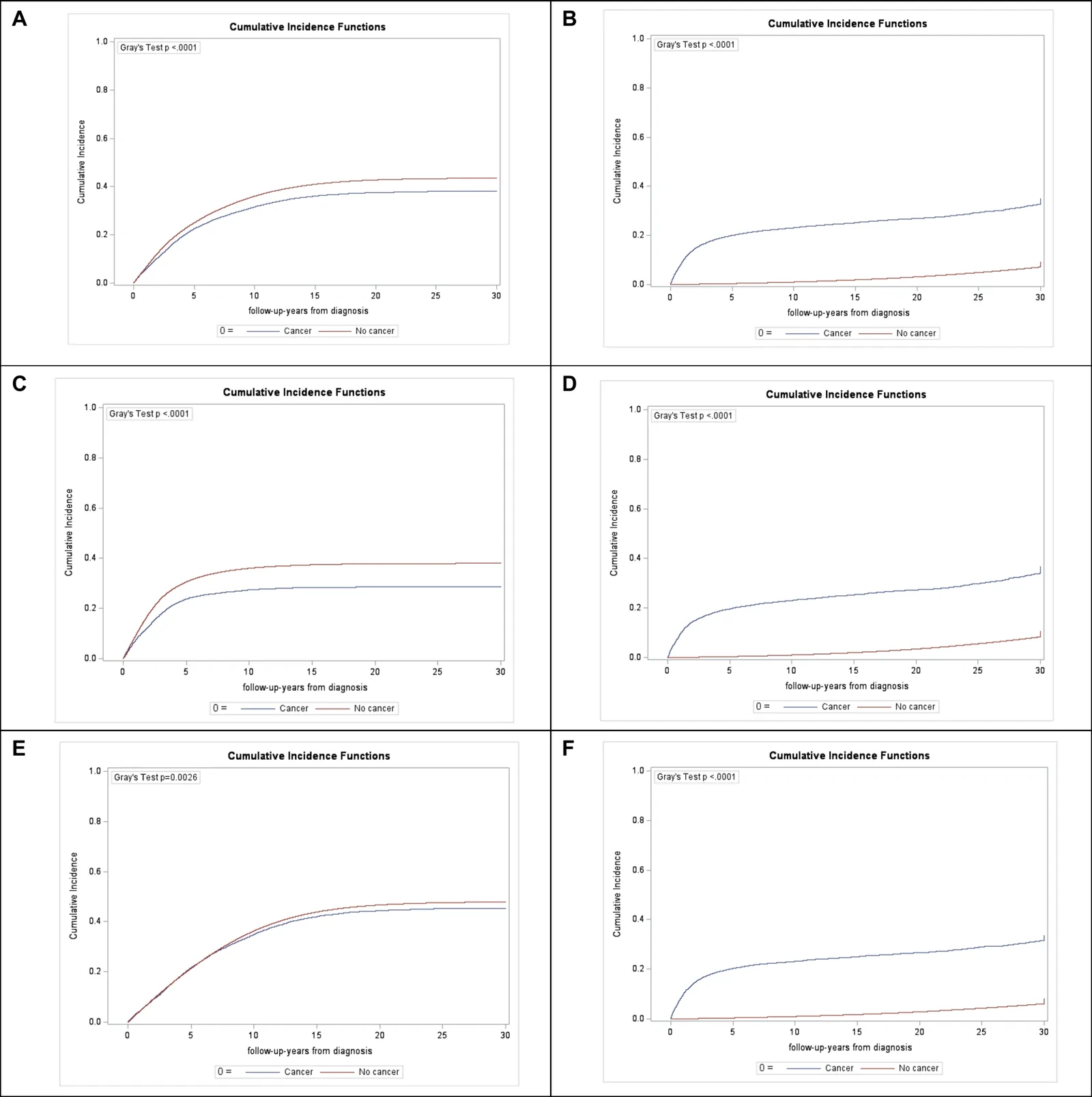
Cumulative incidence curves for fatherhood (left **A**, **C**, **E**) and death (right **B**, **D**, **F**) after diagnosis or study entry among all men, men with one or more children before diagnosis or study entry, and men without children at diagnosis or study entry. Follow-up years are from the time of diagnosis or study entry

**Fig. 2:**
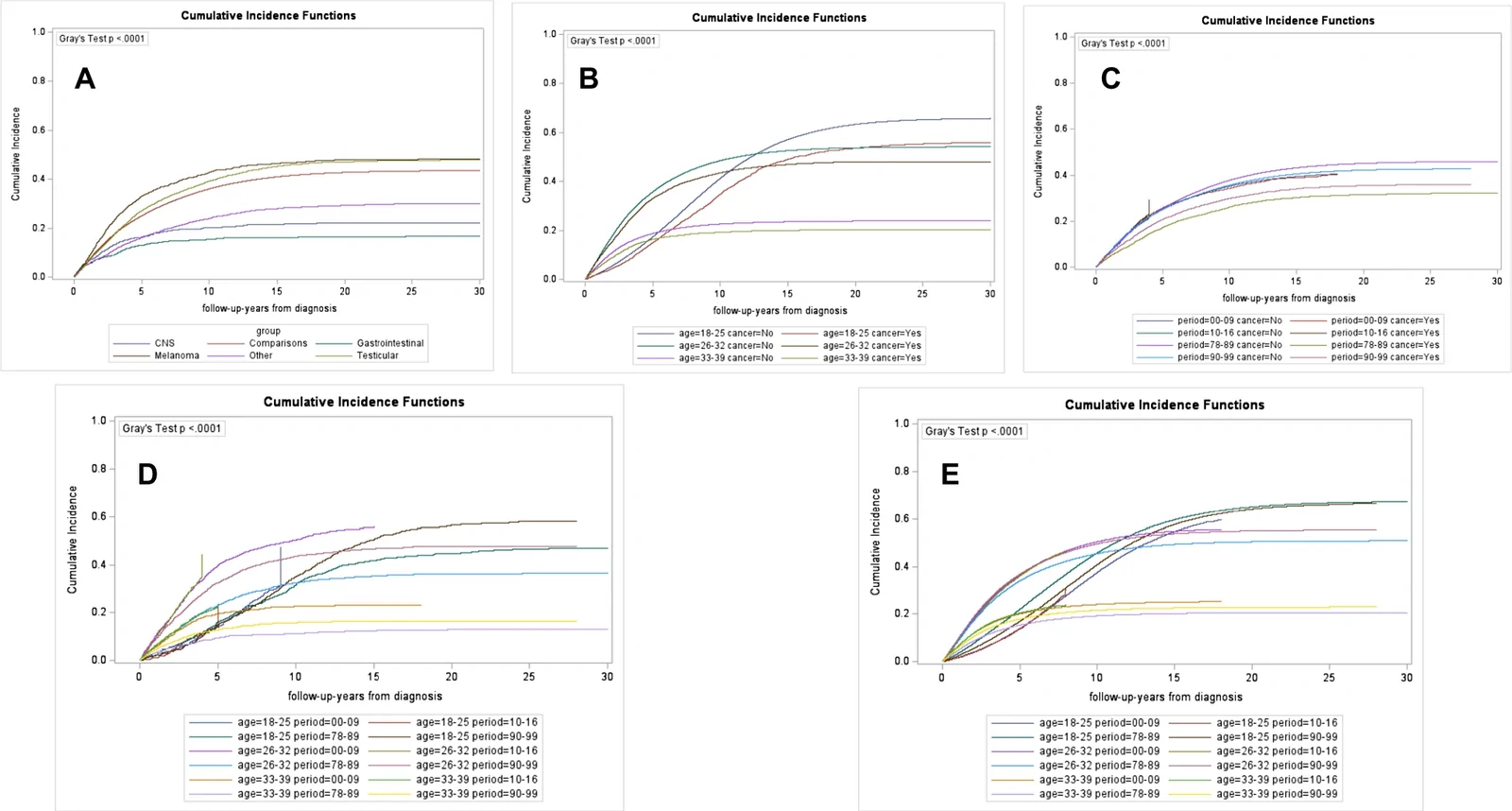
Cumulative incidence curves for fatherhood after diagnosis among men diagnosed with cancer when aged 18–39 compared undiagnosed comparisons stratified by **A** cancer groups (leukemia, lymphoma, CNS, gastrointestinal, testicular cancer, malignant melanoma, other cancers) and the comparisons; **B** age at diagnosis or study entry (age intervals 18–25, 26–32, 33–39); **C** year of diagnosis or study entry (year intervals 1978–1989, 1990–1999, 2000–2009, 2010–2016). The cumulative incidence curves for fatherhood are stratified by the combination of year of diagnosis or study entry (year intervals 1978–1989, 1990–1999, 2000–2009, 2010–2016) and age at diagnosis or study entry (age intervals 18–25, 26–32, 33–39) among **D** men diagnosed with cancer; and **E** the undiagnosed comparisons. Follow-up years are from the time of diagnosis or study entry. *CNS* central-nerve system

The cumulative incidence of fatherhood and death during follow-up, accounting for death as a competing event, is shown in cumulative incidence curves (Figs. 1 and 2). These curves illustrate the cumulative incidence of fatherhood and death among men with and without children at diagnosis (Fig. 1), and the cumulative incidence of fatherhood across different cancer groups, age intervals, and time periods as well as separately for men with cancer and comparisons grouped by age intervals and time periods (Fig. 2). The cumulative incidence of death across cancer groups, age intervals, and grouped by age intervals and time periods is found in Supplementary 4.

The cumulative incidence of fatherhood after diagnosis was lower for men with cancer compared to the comparisons (0.40 [95% CI 0.37–0.38] vs. 0.43 [95% CI 0.43–0.43], respectively) (Fig. 1A). Among men who already had one or more children before diagnosis, the cumulative incidence of fatherhood was lower than that of comparisons without children at study entry (Fig. 1C). In contrast, for men without children before diagnosis, the cumulative incidence of fatherhood was notably higher and closely aligned with that of childless comparisons (Fig. 1E). The cumulative incidence of fatherhood was highest among those with malignant melanoma and testicular cancer and lowest among those with gastrointestinal and CNS cancer (Fig. 2A). Due to limited cases and events, cumulative incidence for men with lymphoma and leukemia could not be visualized in Fig. 2.

Mortality was low among men without cancer, whereas men diagnosed with cancer and in particular those with CNS cancer, gastrointestinal cancer, or leukemia showed a cumulative incidence of death between approximately 0.40 and 0.65 (Supplementary 4.A). Due to limited cases and events, cumulative incidence for men with lymphoma could not be visualized in Supplementary 4. Across all cancer groups, the incidence of death plateaued over time. An older age at diagnosis (33–39 years) was associated with an increased cumulative incidence of death (Supplementary 4.B), and similarly, diagnosis in earlier periods was linked to higher mortality (Supplementary 4.C). In younger age groups, differences in follow-up time due to the end of the study period appeared to “interrupt” individuals during their reproductive years, as cumulative incidence curves had not yet plateaued by the end of follow-up for both diagnosed and undiagnosed men (Supplementary 4.D and 4.E).

### The probability of fatherhood after diagnosis

Table 2 presents the unadjusted and adjusted HRs (aHR) of fatherhood after diagnosis or study entry.

**Table 2:** The probability of fatherhood after cancer, follow-up time, and unadjusted and adjusted HRs of achieving fatherhood after cancer for all men with cancer (n = 16,913); men with one or more children before diagnosis (n = 7239); and men without children before diagnosis (n = 9,674). Adjusted HRs were stratified by age at diagnosis. HR was adjusted for year of diagnosis (or study entry for the comparisons) and immigration status. In analyses including the total study population, the HR was also adjusted for children at diagnosis for men with cancer and at study entry among comparisons. IQR interquartile range, HR hazard ratio, CNS central-nerve system

Cancer type	Median follow-up time after diagnosis or study entry, years (IQR)	Men who achieved fatherhood after cancer or study entry, ***N*** (%)	Time from diagnosis until fatherhood after cancer Median years (IQR)	Unadjusted HR (95% CI)	Adjusted HR (95% CI)			
				All	All	18–25 years	26–32 years	33–39 years
All (***n*** = 16,913)								
All cancers	4.9 (1.7–12.9)	5474 (32)	3.3 (1.5–6.5)	1.05 (1.03–1.09)	0.88 (0.86–0.91)	0.85 (0.81–0.89)	0.88 (0.84–0.91)	0.92 (0.87–0.97)
Leukemia	2.6 (1.0–7.7)	179 (21)	2.7 (1.1–8.6)	0.95 (0.82–1.10)	0.80 (0.70–0.93)	0.76 (0.58–0.99)	0.80 (0.64–1.02)	0.82 (0.62–1.08)
Lymphoma	4.6 (2.4–8.1)	188 (25)	5.0 (2.3–5.0)	1.01 (0.87–1.16)	0.77 (0.67–0.89)	0.86 (0.66–1.13)	0.74 (0.59–0.93)	0.76 (0.59–0.98)
CNS	2.6 (1.0–6.9)	218 (20)	2.7 (1.1–7.0)	0.90 (0.78–1.02)	0.77 (0.67–0.88)	0.60 (0.46–0.77)	0.88 (0.72–1.07)	0.78 (0.61–1.01)
Gastrointestinal	1.8 (0.6–6.4)	191 (15)	2.0 (0.6–8.4)	0.91 (0.79–1.05)	0.77 (0.67–0.89)	1.13 (0.80–1.59)	0.66 (0.52–0.83)	0.74 (0.60–0.92)
Testicular	7.6 (2.9–17.8)	2654 (41)	9.1 (2.7–21.7)	1.04 (1.01–1.08)	0.87 (0.84–0.91)	0.84 (0.79–0.90)	0.86 (0.81–0.91)	0.95 (0.87–1.04)
Melanoma	4.8 (2.1–10.6)	911 (40)	5.2 (2.1–13.4)	1.51 (1.41–1.61)	1.22 (1.14–1.30)	1.18 (1.03–1.36)	1.22 (1.11–1.34)	1.22 (1.09–1.37)
Other	4.3 (1.2–13.5)	1133 (27)	4.1 (1.1–15.7)	0.92 (0.87–0.98)	0.80 (0.76–0.85)	0.80 (0.73–0.88)	0.79 (0.72–0.86)	0.78 (0.69–0.88)
Comparisons	8.2 (3.1–17.8)	497,261 (38)	3.3 (1.4–6.7)	1	1	1	1	1
Men with a one or more children before diagnosis (***n*** = 7239)								
All cancers	4.9 (1.6–15.7)	1926 (26)	2.1 (0.9–3.7)	0.83 (0.79–0.87)	0.84 (0.81–0.88)	0.81 (0.67–0.98)	0.84 (0.78–0.89)	0.85 (0.79–0.91)
Leukemia	2.7 (1.1–8.6)	69 (19)	2.1 (0.7–3.8)	0.78 (0.61–0.98)	0.76 (0.60–0.97)	0.95 (0.40–2.31)	0.86 (0.61–1.20)	0.61 (0.42–0.89)
Lymphoma	5.2 (2.3–9.1)	62 (24)	2.1 (0.9–3.3)	0.80 (0.62–1.02)	0.63 (0.49–0.80)	0.41 (0.10–1.64)	0.64 (0.45–0.91)	0.65 (0.45–0.93)
CNS	2.7 (1.1–7.0)	97 (20)	1.1 (0.6–2.9)	0.79 (0.64–0.96)	0.80 (0.66–0.98)	0.97 (0.43–2.15)	0.85 (0.65–1.12)	0.71 (0.52–0.97)
Gastrointestinal	2.0 (0.6–8.4)	91 (14)	1.4 (0.6–3.5)	0.70 (0.57–0.86)	0.69 (0.56–0.85)	0.28 (0.04–1.95)	0.74 (0.53–1.02)	0.65 (0.50–0.86)
Testicular	9.1 (2.7–21.7)	819 (33)	2.3 (1.0–3.9)	0.78 (0.73–0.84)	0.82 (0.76–0.88)	0.74 (0.57–0.96)	0.80 (0.73–0.87)	0.89 (0.80–0.99)
Melanoma	5.2 (2.1–13.4)	384 (33)	2.0 (1.0–3.2)	1.18 (1.07–1.30)	1.14 (1.03–1.26)	1.09 (0.60–1.97)	1.15 (0.99–1.34)	1.11 (0.96–1.27)
Other	4.1 (1.1–15.7)	404 (22)	2.2 (0.8–4.1)	0.76 (0.69–0.84)	0.80 (0.73–0.88)	0.96 (0.68–1.37)	0.78 (0.68–0.91)	0.77 (0.67–0.89)
Comparisons	8.5 (2.5–21.0)	190,550 (35)	2.0 (1.0–3.8)	1	1	1	1	1
Men without children before diagnosis (n = 9674)								
All cancers	4.9 (1.8–11.4)	3548 (36)	4.4 (2.1–8.0)	1.18 (1.14–1.22)	0.90 (0.87–0.93)	0.86 (0.82–0.90)	0.90 (0.85–0.94)	1.03 (0.94–1.13)
Leukemia	2.6 (1.0–7.5)	110 (22)	4.0 (1.8–9.3)	1.03 (0.85–1.24)	0.80 (0.66–0.97)	0.75 (0.57–0.99)	0.72 (0.52–0.99)	1.25 (0.83–1.91)
Lymphoma	4.5 (2.4–7.4)	126 (26)	3.5 (2.0–5.6)	1.13 (0.95–1.35)	0.85 (0.72–1.02)	0.91 (0.69–1.19)	0.79 (0.59–1.06)	0.91 (0.63–1.31)
CNS	2.5 (0.9–6.8)	121 (21)	2.6 (1.4–5.9)	0.91 (0.76–1.09)	0.71 (0.60–0.86)	0.58 (0.44–0.76)	0.83 (0.63–1.10)	0.94 (0.60–1.48)
Gastrointestinal	1.7 (0.5–5.4)	100 (17)	2.7 (0.7–5.3)	1.05 (0.86–1.27)	0.79 (0.65–0.96)	1.22 (0.86–1.73)	0.58 (0.42–0.81)	0.86 (0.60–1.24)
Testicular	7.0 (3.1–15.0)	1835 (46)	4.6 (2.4–8.4)	1.20 (1.14–1.25)	0.90 (0.86–0.94)	0.86 (0.80–0.92)	0.91 (0.85–0.97)	1.06 (0.92–1.21)
Melanoma	4.5 (2.1–8.6)	527 (48)	3.5 (1.6–6.1)	1.71 (1.57–1.86)	1.28 (1.17–1.39)	1.21 (1.04–1.40)	1.25 (1.10–1.41)	1.56 (1.27–1.91)
Other	4.4 (1.2–12.2)	729 (30)	5.7 (2.6–9.6)	1.02 (0.95–1.10)	0.79 (0.74–0.85)	0.80 (0.72–0.88)	0.78 (0.69–0.88)	0.77 (0.63–0.96)
Comparisons	8.0 (3.5–17.2)	306,711 (38)	4.7 (2.1–8.3)	1	1	1	1	1

The aHR for achieving fatherhood after diagnosis was lower among men with cancer (0.88 [95% CI 0.86–0.91]), revealing a lower probability of fatherhood compared to undiagnosed comparisons. The probability of fatherhood following diagnosis varied significantly across cancer groups and increased with age at diagnosis (from 0.85 [95% CI 0.81–0.89] to 0.92 [95% CI 0.87–0.97]).

Young men with malignant melanoma had the highest aHRs, with a significantly higher probability of fathering a child post-diagnosis (1.22 [95% CI 1.14–1.30]), followed by a slightly lower aHR for men with testicular cancer (0.87 [95% CI 0.84–0.91]) compared with the comparisons. Men with lymphoma, CNS, and gastrointestinal cancers had the lowest aHRs of fathering a child (lymphoma, 0.77 [95% CI 0.67–0.89]; CNS, 0.77 [95% CI 0.67–0.88]; gastrointestinal, 0.77 [95% CI 0.67–0.89]) among the cancer groups.

Among men who had one or more children before diagnosis, the aHR for having an additional child post-diagnosis was lower compared to the comparison group (0.84 [95% CI 0.81–0.88]). Within this group, 1926 men (26%) became fathers after their diagnosis, with a median age at diagnosis of 34.9 years. The median follow-up time until fatherhood after cancer was 2.1 years (IQR 0.9–3.7; min–max, 0–22 years). For men without children at the time of diagnosis, the aHR of fathering a child post-diagnosis was slightly higher than for those who already had children at diagnosis (0.90 [95% CI 0.87–0.93]), yet still lower than the comparison group. Among these men, 3548 (36%) became fathers after diagnosis, with a lower median age at diagnosis (28.1 years) compared to men who already had children. Additionally, men without children at diagnosis had a longer median follow-up time (4.4 years; IQR 2.1–8.0; min–max, 0–27 years) before fathering their first child.

### Trends over time in the probability of achieving fatherhood after diagnosis

Sensitivity analyses with 5-year intervals did not show any differences related to the chosen periods (1978–1989, 1990–1999, 2000–2009, 2010–2016).

**Fig. 3:**
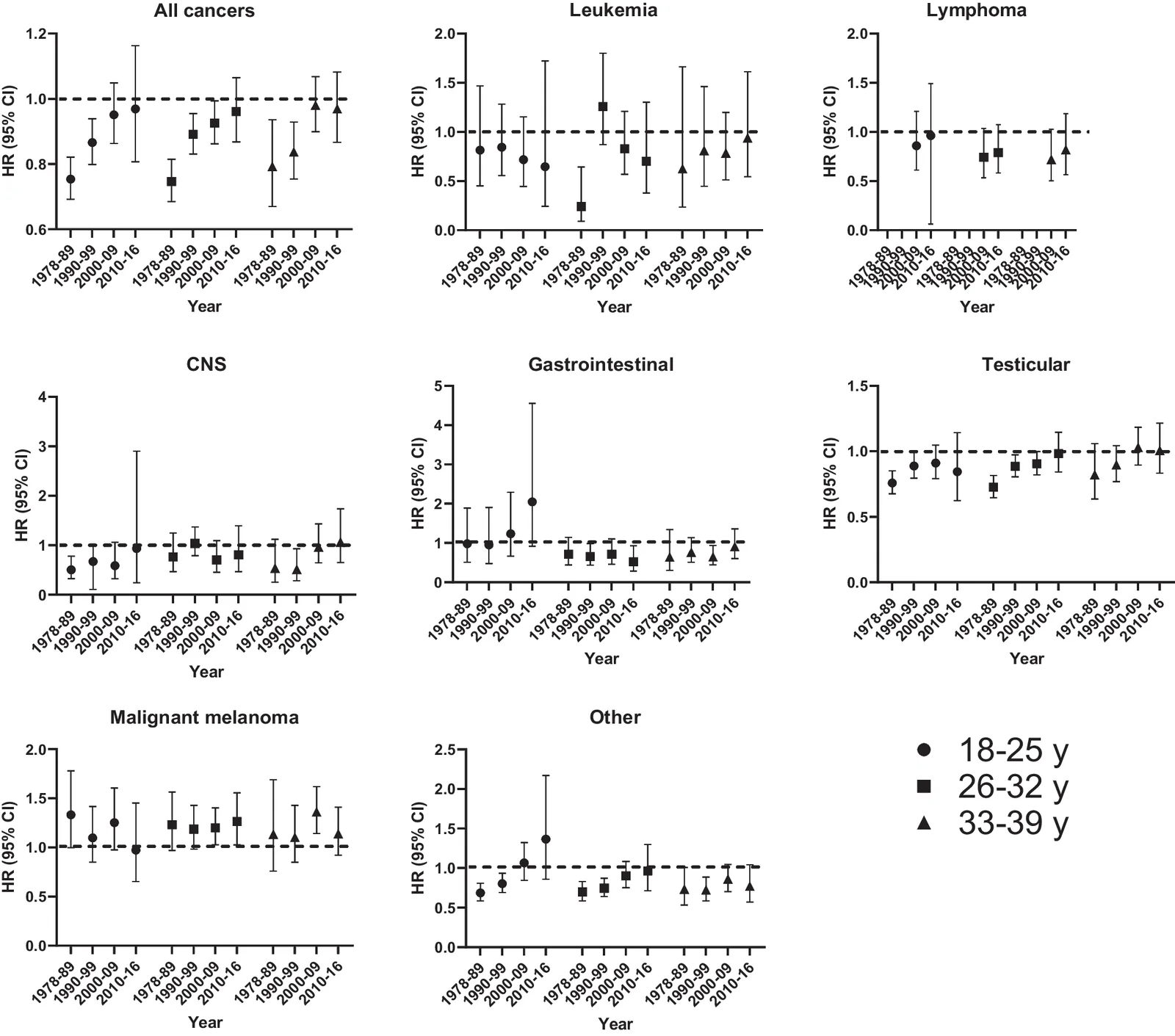
Trends over time for adjusted HR of fatherhood after cancer, stratified by cancer groups and age intervals. The age intervals were divided into 18–25, 26–32, and 33–39 years. The year intervals were divided into 1978–1989, 1990–1999, 2000–2009, and 2010–2016. HR was adjusted for children before baseline, year of diagnosis or study entry*,* and immigration status. *CNS* central-nerve system

Figure 3 illustrates the trends in the aHRs for fatherhood, stratified by cancer groups, age at diagnosis, and time periods. Across all cancer groups, the probability of becoming a father after a cancer diagnosis demonstrated a general trend of improvement over time, particularly in the later time periods. Men with malignant melanoma consistently exhibited the highest aHRs across all time periods. Primarily, among men with testicular cancer and “other cancers,” an apparent rise in aHRs in all age intervals was found with increased time periods. This trend was also recognized in men with CNS cancer, especially within the two latter periods. In contrast, men with gastrointestinal cancers, leukemia, and lymphoma consistently showed lower aHRs of fatherhood with only slight improvements over time.

### The cumulative incidence and aHR of partnership formation after diagnosis or study entry

**Fig. 4:**
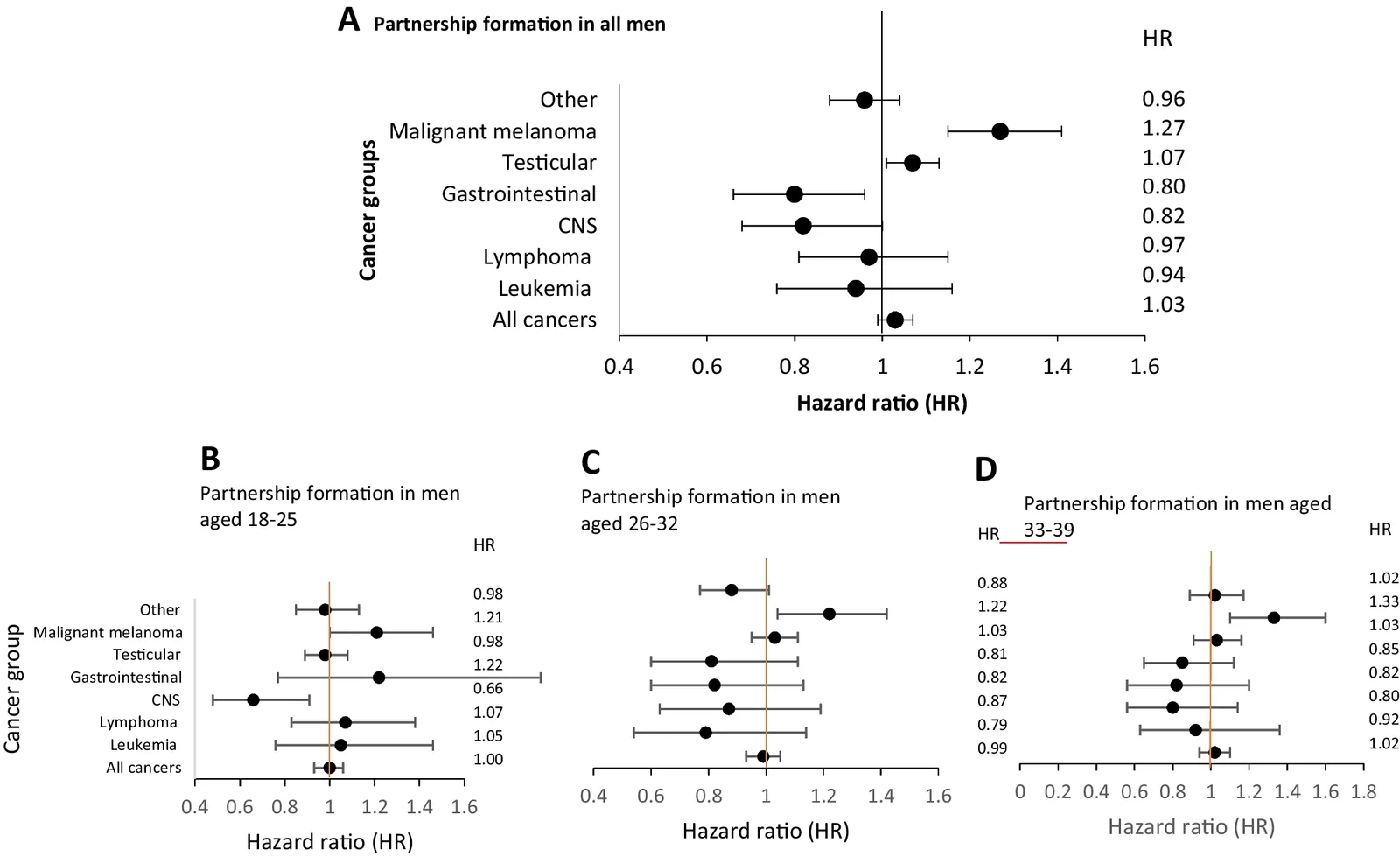
Forest plots of hazard ratios (HR) for partnership formation after diagnosis among men registered as single at diagnosis. **A** HR with 95% CIs of partnership formation for all single men diagnosed with cancer (*n* = 5,725), stratified into cancer groups, with undiagnosed comparisons as reference (*n* = 365,351). **B** HR with 95% CIs of partnership formation for single men diagnosed with cancer when aged 18–25 (*n* = 1459), with comparisons as reference (*n* = 88,816). **C** HR with 95% CIs of partnership formation for single men diagnosed with cancer when aged 26–32 (*n* = 2135), with comparisons as reference (*n* = 135,953). **D** HR with 95% CIs of partnership formation for single men diagnosed with cancer when aged 33–39 (*n* = 2131), with comparisons as reference (*n* = 140,582). The horizontal lines through the circles represent the 95% CI; ongoing lines without a bracket indicate upper CI above 1.6. All analyses are adjusted for age at diagnosis and immigration status. CNS, central-nerve system

Table 3. shows the cumulative incidences as well as the unadjusted HR and aHRs of partnership formation after diagnosis or study entry. The HRs were adjusted for age at diagnosis and immigration status. The cumulative incidence of partnership formation after diagnosis or study entry was lower among men with cancer compared to the comparisons (0.58 [95% CI 0.56–0.60] vs. 0.72 [95% CI 0.72–0.72]). Figure 4 presents forest plots showing aHR of partnership formation after diagnosis or study entry for men who were single at diagnosis or study entry. The aHR for partnership formation following a cancer diagnosis was comparable to that of comparisons (1.03 [95% CI 0.99–1.07]). However, differences emerged across cancer groups. Among men with gastrointestinal and CNS cancers, we found a trend towards a lower aHR of partnership formation compared to comparisons (0.80 [95% CI 0.66–0.96] and 0.82 95% [CI 0.68–1.00], respectively). Additionally, the probability of partnership formation decreased with increased age at diagnosis for those with lymphoma, CNS, and gastrointestinal cancers.

**Table 3:** Cumulative incidences and unadjusted and adjusted HR of partnership formation after diagnosis or study entry among men who were single at diagnosis or study entry. The adjusted HR was stratified into age intervals at diagnosis and cancer group. The HRs were adjusted for age at diagnosis and immigration status; ¤15-year cumulative incidence. ^#^30-year cumulative incidence. *CNS* central-nerve system

Cancer type	The cumulative incidence of partnership formation after diagnosis or study entry (95% CI)^#^	Unadjusted HR for partnership formation after diagnosis (95% CI)	Adjusted HR for partnership formation after diagnosis (95% CI)
All cancers	0.58 (0.56–0.60)	1.03 (0.99–1.07)	1.03 (0.99–1.07)
Leukemia	0.44 (0.36–0.51)	0.93 (0.76–1.15)	0.94 (0.76–1.16)
Lymphoma	0.60 (0.52–0.67)¤	0.94 (0.79–1.11)	0.97 (0.81–1.15)
CNS	0.34 (0.29–0.39)	0.82 (0.68–1.10)	0.82 (0.68–1.00)
Gastrointestinal	0.31 (0.26–0.36)	0.79 (0.66–0.96)	0.80 (0.66–0.96)
Testicular	0.71 (0.68–0.73)	1.07 (1.01–1.13)	1.07 (1.01–1.13)
Melanoma	0.73 (0.68–0.77)	1.26 (1.14–1.39)	1.27 (1.15–1.41)
Other	0.40 (0.44–0.51)	0.97 (0.89–1.05)	0.96 (0.88–1.04)
Comparisons	0.72 (0.72–0.72)	1	1

## Discussion

This study offers new insights into the probability of achieving fatherhood and partnership formation following a cancer diagnosis among Danish men aged 18–39, examining variations across different cancer types, age groups, and periods.

The lower proportion of immigrants and second-generation immigrants among men diagnosed with cancer, compared with the age-matched controls, is most likely driven by the younger age structure and shorter cumulative exposure to Danish lifestyle-related cancer risks in these populations; by contrast, educational and partnership differences largely mirror the social-gradient pattern seen in cancer incidence more generally and did not materially influence the associations under study.

Regarding fatherhood, our study found a lower probability after diagnosis than the comparisons. This aligns with previous research. Gunnes et al. (2016) reported significantly lower HR of fatherhood (0.72 [95% CI 0.68–0.76]) among men diagnosed under age 25 compared to comparisons [27]. In our study, we observed a slightly higher adjusted HR of fatherhood for men diagnosed at 18–25 years (0.85 [95% CI 0.81–0.89]), suggesting a relatively better probability of fatherhood for young adult cancer survivors compared to those also diagnosed in childhood and adolescence. Our results also support findings by Syse et al. (2007) and Armuand et al. (2017), who reported lower fatherhood rates across cancer types, particularly in those with gastrointestinal and CNS cancers [23, 28]. Also, our results confirm findings by Sylvest et al. (2021), who analysed 9353 Danish males diagnosed with cancer between ages 0 and 30. Among those surviving into adulthood, lower HR for fatherhood was found among those with CNS cancers (0.67 [95% CI 0.57–0.79]) and hematological cancers (0.90 [95% CI 0.81–1.01]) [30].

Our findings indicate that men who were single at diagnosis generally formed partnerships at rates comparable to age-matched comparisons (overall aHR ≈ 1.0). The notable exceptions were men diagnosed with gastrointestinal cancer (aHR 0.80, 95% CI 0.76–0.85) or CNS cancer (aHR 0.82, 95% CI 0.78–0.86). These lower hazard ratios were particularly evident in the 26–32 and 33–39 year age intervals, underscoring that the deficit intensifies in later young adulthood. Gunnes et al. (2016) and Sylvest et al. (2022) reported lower marriage rates for males diagnosed with cancer; however, in their population, the lower rates were especially at ages below 25 and 30 years, respectively, with CNS cancer survivors experiencing a significant reduction [27, 31]. Similarly, Rabin et al. (2019) observed lower odds of marriage or cohabitation and delays in romantic relationships among young cancer survivors aged 18–44 at diagnosis [9].

Several factors may impact the lower findings of fatherhood and partnership formation among those with CNS and gastrointestinal cancers. In our study, men with CNS and gastrointestinal cancers had the highest 5-year cumulative incidences of death after diagnosis (Supplementary 4.A.). Because an elevated risk of early mortality effectively shortens the reproductive window, young adults with poor prognosis may have fewer practical opportunities to form stable partnerships and attempt conception. Moreover, anticipating a limited life expectancy can discourage partner-seeking or fatherhood plans altogether, as some individuals prefer to avoid burdening a future partner or leaving potential children without a parent [41, 42]. CNS cancer treatments involving surgery, chemotherapy, and radiotherapy may result in lasting cognitive and physical impairments, which can hinder engagement in romantic relationships and family building [43, 44]. However, cognitive impairments are not limited to CNS cancers, as they also occur in non-CNS cancers, impacting quality of life and role functioning [45]. For men with gastrointestinal cancers, factors such as a change in body image, self-esteem issues due to abdominal scars or an ostomy, and the risk of incontinence may impact partnership formation and sexual health. Almont et al. (2019) reported that approximately half of the participants diagnosed with cancer experienced reduced sexual desire and difficulty reaching orgasm, while two-thirds reported a decreased frequency of intercourse 2 years after diagnosis [46]. Additionally, Préau et al. (2011) found that upper gastrointestinal tract cancers in men were not associated with a strengthening of romantic relationships within 2 years after diagnosis compared to other diagnoses [47].

We also found lower probabilities of fatherhood in men diagnosed with lymphoma, consistent with findings from Syse et al. (2007), Sylvest et al. (2021), and Armuand et al. (2017) [23, 28, 30]. Similarly, Entrop et al. (2023) reported lower fatherhood HRs among patients aged 18–40 years with Hodgkin’s lymphoma treated with BEACOPP (bleomycin sulfate, etoposide phosphate, doxorubicin hydrochloride (Adriamycin), cyclophosphamide, vincristine sulfate (Oncovin), procarbazine hydrochloride, and prednisone) and those with aggressive non-Hodgkin’s lymphoma subtypes compared to matched controls [48, 49]. These lower HRs of fatherhood may be attributed to treatment-related toxicities, cancer activity, and changes in reproductive behavior [50–52].

Contrastingly, young men diagnosed with malignant melanoma or testicular cancer had fatherhood rates close to or higher than the comparisons in our study, which corroborate with findings by Tang et al. (2016) [24]. Our results also align with Bandak et al. (2022), who detailed the impact of specific testicular cancer treatment on fatherhood probabilities. They found lower HRs of fatherhood following combination treatment with bleomycin, etoposide, and cisplatin (BEP) (0.87 [95% CI 0.78–0.97]) and BEP with surgery (0.74 [95% CI 0.63–0.87]), while radiotherapy and surveillance after surgery had less impact [53]. Interestingly, Kim et al. (2010) found that testicular cancer survivors were more susceptible to fertility distress despite similar fatherhood rates compared to comparisons [54], suggesting that oncofertility counseling also is important in this cancer group to prevent fertility-related distress.

Our findings suggest that men without children before diagnosis were more likely to become fathers afterward; however, they had a more extended follow-up period until fatherhood compared to those who already had children at diagnosis. Our results align with previous studies by Magelssen et al. (2008) and Tang et al. (2016), which reported similar trends across various cancer diagnoses [24, 55]. Nonetheless, both groups had a lower probability of fatherhood than their respective comparisons. The longer interval to fatherhood seen in men who were childless at diagnosis likely reflects their younger median age. Older men who already had children had often finished building their families, whereas younger, childless men—still seeking partners and monitored for a longer period—enjoyed a wider reproductive window and thus more opportunities to become fathers after cancer[56].

In general, we observed an increased probability of fatherhood over time. Likewise, Syse et al. (2007) found that the predicted ORs for live births among survivors diagnosed at 17–44 years improved from 0.60 in 1965 to 0.85 in 2001 [23]. Sylvest et al. (2021) also reported increasing adjusted HRs of fatherhood between 1978 and 2016 (from 0.72 [95% CI 0.68–0.77] to 1.06 [95% CI 0.95–1.19]) [30]. Our findings, along with previous studies, suggest that improvements in cancer diagnostics, treatments, and fertility preservation—along with less restrictive views on fatherhood after cancer due to the risk of birth abnormalities—have increased fatherhood rates, though they remain lower than in men without cancer [20, 57–59]. The shift in the adjusted hazard ratios (HRs) from 1990–1994 to 1995–1999 seen in the sensitivity analyses with 5-year intervals is explained by the introduction of systematic ART treatment in Denmark from 1994 onwards [60].

While it is well established that cancer and its treatments can impact fertility, other factors may also influence parenthood [7, 19–22]. Beyond physical challenges, concerns about disease recurrence and the health of future offspring may reduce the desire to have children [61, 62]. Lehmann et al. (2024) reported that 7.6% of 1457 men across various cancer types did not want to have first or additional children due to their cancer [63]. This suggests that fatherhood and partnership formation may be affected even among survivors with a more optimistic prognosis.

Psychological and social factors may also play a role in shaping fatherhood and partnership formation after cancer. Young men may encounter social and cultural challenges that affect their sense of masculinity and identity [15] while also being less likely than women to discuss fertility concerns and seek support openly [64]. Furthermore, uncertainty about when and how to address fertility or sexual health issues with a (new) partner may further impact the pursuit of fatherhood [11, 65].

## Strengths and limitations

This is a national register-based study based on the DANAC II cohort, which inherently limits access to certain factors that may influence outcomes, such as specific cancer treatment details, fertility preservation efforts before diagnosis, fertility treatment after cancer treatment, and personal intentions regarding parenthood and family formation after diagnosis. Despite these limitations, the study offers substantial and systematic sociodemographic background information. A significant strength is the extensive population size, comprising over 1.3 million participants nationwide across four decades, with near-complete follow-up. This large, comprehensive population and consistent and mandatory cancer registration in all Danish hospitals significantly reduce the risk of systematic selection and misclassification. Additionally, the data was collected independently of the study hypothesis, further minimizing the potential for systematic or differentiated misclassification.

A limitation is that pre-diagnosis parenthood was coded only as none versus ≥ 1 child; we did not apply a multi-process model that tracks individual parity transitions (0 → 1, 1 → 2, ≥ 2), which may have obscured subtler differences in post-diagnosis fertility trajectories. Furthermore, partnership formation and live birth were modelled separately (each with death as a competing risk); a full multi-state model that jointly captures transitions between relationship and parenthood states was not implemented with the current data but represents an important aspect for future research.

It must also be mentioned that it would have been relevant to include data within recent years. However, follow-up ended on 31 December 2017 because registry linkage in the DANAC II cohort is currently complete only through that date, and data for subsequent years are not yet available. Furthermore, because some registry variables from the initial study period (1978–1985) were missing and assumed to be missing at random, a degree of residual bias due to incomplete data cannot be completely ruled out.

Temporal trends were explored with decade-stratified models. However, residual confounding by more granular shifts in social norms and medical practice within each decade cannot be entirely excluded.

Because educational attainment and partnership status were treated as mediators and therefore not included in the primary adjustment set, some residual confounding by these factors cannot be entirely ruled out.

Excluding men whose partners were pregnant at the index date may modestly underestimate the true impact of cancer on fatherhood; however, this conservative approach avoids inflating post-diagnosis fertility rates with conceptions that occurred before diagnosis and cancer treatment.

Finally, cancer sites were combined into broad groups to secure stable estimates; this pragmatic pooling (e.g., thyroid cancers and sarcomas within “other solid tumors” and pancreatic with colon cancers in the GI group) may mask prognosis-specific differences and could influence the observed mortality and fertility patterns. Future research could enable more finely stratified analyses nuancing these aspects.

## Clinical implications

The patterns observed in our study suggest that the impact of a cancer diagnosis on social and reproductive outcomes varies substantially by cancer type and age at diagnosis. Although the probability of fatherhood and partnership formation after diagnosis more closely resembles that of the comparisons when compared to young women with cancer [66], qualitative studies indicate that partnership formation and fertility are often of great concern among men diagnosed with cancer in their reproductive years [9, 15, 64, 67]. They may experience long-term fertility sequelae after diagnosis [19]. This, along with our findings of lower probability of fatherhood, highlights the need for and importance of oncofertility counseling and timely fertility preservation referrals for young men diagnosed with cancer. Additionally, tailored follow-up care during their cancer trajectory is important [68, 69], particularly for those diagnosed at a younger age, and those with lymphoma, gastrointestinal, and CNS cancers. Future research should employ multistate or other longitudinal modelling approaches to trace the dynamic interplay between partnership formation and parenthood after cancer. In parallel, implementation-science studies are needed to test the real-world uptake and effectiveness of national oncofertility guidelines for young male survivors.

## Conclusion

The probabilities of fatherhood were generally lower among men diagnosed with cancer, varying by cancer groups, age, and periods, with younger men and certain cancer groups facing the most significant challenges. Encouragingly, fatherhood rates improved over time. Notably, single men at diagnosis had similar partnership probabilities as comparisons, although those with gastrointestinal or CNS cancers faced greater difficulties. However, the lower probability of fatherhood underscores the need for and importance of oncofertility counseling, timely fertility preservation referrals, and tailored follow-up care, considering both cancer groups and age at diagnosis.

## Supplementary Information

Below is the link to the electronic supplementary material.

ESM 1 (DOCX 194 KB)

ESM 2 (DOCX 195 KB)

## Data Availability

No datasets were generated or analysed during the current study.
