# Supplementary material for: Partnership and fatherhood outcomes in young men diagnosed with cancer: a nationwide register-based study
Source: J Cancer Surviv. 2025 Jun 10;20(Suppl 1):578–92. doi: 10.1007/s11764-025-01849-7 (PMC13676523; doi:10.1007/s11764-025-01849-7)
Supplement: Supplementary file 1 [file 11764_2025_1849_MOESM1_ESM.docx]

Supplementary 1. The cancer diagnoses are coded by the International Classification of Diseases (ICD-10) and stratified into the following categories: leukemia, lymphoma, CNS cancers, gastrointestinal (GI) cancers, malignant melanoma, testicular cancer, and other solid tumors (e.g., sarcomas, thyroid, lung, and urothelial cancers).

| **Categorization** | **ICD 10 codes** | **Definition of malignant neoplasm** |
| --- | --- | --- |
| **Leukemia** | C90.0-90.9 | Multiple myeloma and malignant plasma cell neoplasms |
|  | C91.0-91.9 | Lymphoid leukemia |
|  | C92.0-92.9 | Myeloid leukemia |
|  | C93.0-93.9 | Monocytic leukemia |
|  | C94.0-94.9 | Other leukemias of specified cell types |
|  | C95.0-95.9 | Leukemia of unspecified cell type |
|  | C96.0-96.9 | Other and unspecified malignant neoplasms of lymphoid, hematopoietic and related tissue |
| **Lymphoma** | C81.0-81.9 | Hodgkin lymphoma |
|  | C82.0-82.9 | Follicular lymphoma |
|  | C83.0-83.9 | Non-follicular lymphoma |
|  | C84.0-84.9 | Mature T/NK-cell lymphomas |
|  | C85.0-85.9 | Other and unspecified types of non- |
|  | C86.0-86.9 | Other specified types of T/NK-cell lymphoma |
|  | C88.0-88.9 | Malignant immunoproliferative diseases |
| **CNS cancer** | C70.0-70.9 | Malignant neoplasm of meninges |
|  | C71 | Malignant neoplasm of brain |
|  | C72 | Malignant neoplasm of spinal cord, cranial nerves, and other parts of central nervous system |
|  | D42 | Neoplasm of uncertain or unknown behavior of meninges |
|  | D43 | Neoplasm of uncertain or unknown behavior of brain and central CNS |
| **Testes** | C62 | Malignant neoplasm of testis |
| **Melanoma** | C43 | Malignant melanoma of skin |
| **GI** | C15.0-15.9 | Malignant neoplasm of esophagus |
|  | C16.0-16.9 | Malignant neoplasm of stomach |
|  | C17.0-17.9 | Malignant neoplasm of small intestine |
|  | C22.0-22.9 | Malignant neoplasm of liver and intrahepatic bile ducts |
|  | C23 | Malignant neoplasm of gallbladder |
|  | C24.0-24.9 | Malignant neoplasm of other and unspecified parts of biliary tract |
|  | C25.0-25.9 | Malignant neoplasm of pancreas |
|  | C26.0-26.9 | Malignant neoplasm of other and ill-defined digestive organs |
|  | C18.0-18.9 | Malignant neoplasm of colon |
|  | C19 | Malignant neoplasm of rectosigmoid junction |
|  | C20 | Malignant neoplasm of rectum |
|  | C21.0-21.8 | Malignant neoplasm of anus and anal canal |
| **Other** | C00.0-00.9 | Malignant neoplasm of lip |
|  | C01 | Malignant neoplasm of base of tongue |
|  | C02.0-02.9 | Malignant neoplasm of other and unspecified parts of tongue |
|  | C03.0-03.9 | Malignant neoplasm of gum |
|  | C04.0-04.9 | Malignant neoplasm of floor of mouth |
|  | C05.0-05.9 | Malignant neoplasm of palate |
|  | C06.0-06.9 | Malignant neoplasm of other and unspecified parts of mouth |
|  | C07 | Malignant neoplasm of parotid gland |
|  | C08.0-08.9 | Malignant neoplasm of other and unspecified major salivary glands |
|  | C09.0-09.9 | Malignant neoplasm of tonsil |
|  | C10.0-10.9 | Malignant neoplasm of oropharynx |
|  | C11.0-11.9 | Malignant neoplasm of nasopharynx |
|  | C12 | Malignant neoplasm of piriform sinus |
|  | C13.0-13.9 | Malignant neoplasm of hypopharynx |
|  | C14.0-14.8 | Malignant neoplasm of other and ill-defined sites in the lip, oral cavity, and pharynx |
|  | C30.0-30.9 | Malignant neoplasm of nasal cavity and middle ear |
|  | C31.0-31.9 | Malignant neoplasm of accessory sinuses |
|  | C32.0-32.9 | Malignant neoplasm of larynx |
|  | C33 | Malignant neoplasm of trachea |
|  | C34 | Malignant neoplasm of bronchus and lung |
|  | C37 | Malignant neoplasm of thymus |
|  | C38 | Malignant neoplasm of heart, mediastinum and pleura |
|  | C39 | Malignant neoplasm of other and ill-defined sites in the respiratory system and intrathoracic organs |
|  | C40.0-40.9 | Malignant neoplasm of bone and articular cartilage of limbs |
|  | C41.0-41.9 | Malignant neoplasm of bone and articular cartilage of other and unspecified |
|  | C45 | Mesothelioma |
|  | C46 | Kaposi sarcoma |
|  | C47 | Malignant neoplasm of peripheral nerves and autonomic nervous system |
|  | C48 | malignant neoplasm of retroperitoneum and peritoneum |
|  | C49.0-49.9 | Malignant neoplasm of other connective and soft tissue |
|  | C50 | Malignant neoplasm of breast |
|  | C60 | Malignant neoplasm of penis |
|  | C61 | Malignant neoplasm of prostate |
|  | C63 | Malignant neoplasm of other and unspecified male genital organs |
|  | C64 | Malignant neoplasm of kidney, except renal pelvis |
|  | C65 | Malignant neoplasm of renal pelvis |
|  | C66 | Malignant neoplasm of ureter |
|  | C67 | Malignant neoplasm of bladder |
|  | C68 | Malignant neoplasm of other and unspecified urinary organs |
|  | C69 | Malignant neoplasm of eye and adnexa |
|  | C73 | Malignant neoplasm of thyroid gland |
|  | C74 | Malignant neoplasm of adrenal gland |
|  | C75 | Malignant neoplasm of other endocrine glands and related structures |
|  | C76 | Malignant neoplasm of other and ill-defined sites |
|  | C77 | Secondary and unspecified malignant neoplasm of lymph nodes |
|  | C78 | Secondary malignant neoplasm of respiratory and digestive organs |
|  | C79 | Secondary malignant neoplasm of other and unspecified sites |
|  | C80 | Malignant neoplasm, without specification of site |
|  | C97 | Malignant neoplasms of independent (primary) multiple sites |

Supplementary 2. Causal diagram including possible confounders and mediators. A cancer diagnosis is the exposure, fatherhood after cancer is the outcome. Possible confounders/secondary risk factors were year of diagnosis, age at diagnosis, immigration status and having one or more children before diagnosis. Other possible mediators were educational level and partnership status.

Supplementary 3. Causal diagram including possible confounders and mediators. A cancer diagnosis is the exposure, partnership formation after cancer is the outcome. Possible confounders/secondary risk factors were year of diagnosis, age at diagnosis, immigration status and having one or more children before diagnosis. Educational level was a possible mediator.
