# Supplementary material for: Partnership and fatherhood outcomes in young men diagnosed with cancer: a nationwide register-based study
Source: J Cancer Surviv. 2025 Jun 10;20(Suppl 1):578–92. doi: 10.1007/s11764-025-01849-7 (PMC13676523; doi:10.1007/s11764-025-01849-7)
Supplement: Supplementary file 2 [file 11764_2025_1849_MOESM2_ESM.docx]

**A	B**

**C**

A: Due to few cases and events, the cumulative incidence of death for men with lymphoma could not be illustrated. The cumulative incidence of death was truncated at 15 years post-diagnosis for those with leukemia, melanoma, CNS, and Gastrointestinal cancer

**D	E**

D: Due to limited cases and events, the cumulative incidence of death was truncated for those diagnosed/entered the study in 2010–2016 at age 18-25 at 5 years post-diagnosis/study entry

Supplementary 4

Cumulative incidence curves for death after diagnosis among men diagnosed with cancer when aged 18-39 compared undiagnosed comparisons stratified by A) cancer groups (leukemia, lymphoma, CNS, gastrointestinal, testicular cancer, malignant melanoma, other cancers) and the comparisons; B) age at diagnosis or study entry (age intervals 18-25, 26-32, 33-39); C) year of diagnosis or study entry (year intervals 1978-1989, 1990-1999, 2000-2009, 2010-2016). The cumulative incidence is stratified by the combination of year of diagnosis or study entry (year intervals 1978-1989, 1990-1999, 2000-2009, 2010-2016) and age at diagnosis or study entry (age intervals 18-25, 26-32, 33-39) among D) men diagnosed with cancer; and E) the undiagnosed comparisons. Follow-up years is from time of diagnosis or study entry. CNS: central-nerve system.
